# Heat Balance When Climbing Mount Everest

**DOI:** 10.3389/fphys.2021.765631

**Published:** 2021-11-25

**Authors:** Robert K. Szymczak, Krzysztof Błażejczyk

**Affiliations:** ^1^Department of Emergency Medicine, Faculty of Health Sciences, Medical University of Gdańsk, Gdańsk, Poland; ^2^Climate Impacts Laboratory, Institute of Geography and Spatial Organization, Polish Academy of Sciences, Warsaw, Poland

**Keywords:** heat balance, thermal stress, mountain bioclimate, altitude, extremes, mountaineering, Everest

## Abstract

**Background:** Mountaineers must control and regulate their thermal comfort and heat balance to survive the rigors of high altitude environment. High altitudes feature low air pressure and temperatures, strong winds and intense solar radiation, key factors affecting an expedition’s success. All these climatic elements stress human heat balance and survival. We assess components of human heat balance while climbing Mt. Everest.

**Materials and Methods:** We calculated climbers’ heat balance using the Man-ENvironment heat EXchange model (MENEX-2005) and derived meteorological data from the National Geographic Expedition’s *in situ* dataset. Three weather stations sited between 3810 and 7945 m a.s.l. provided data with hourly resolution. We used data for summer (1 May–15 August 2019) and winter (16 October 2019–6 January 2020) seasons to analyze heat balance elements of convection, evaporation, respiration and radiation (solar and thermal).

**Results:** Meteorological and other factors affecting physiology—such as clothing insulation of 3.5–5.5 clo and activity levels of 3–5 MET—regulate human heat balance. Elevation above sea level is the main element affecting heat balance. In summer two to three times more solar radiation can be absorbed at the summit of the mountain than at the foot. Low air pressure reduces air density, which reduces convective heat loss at high altitude by up to half of the loss at lower locations with the same wind speed and air temperature.

**Conclusion:** 1. Alpinists face little risk of overheating or overcooling while actively climbing Mt. Everest, despite the potential risk of overcooling at extreme altitudes on Mt. Everest in winter. 2. Convection and evaporation are responsible for most of the heat lost at altitude. 3. Levels of physical activity and clothing insulation play the greatest role in counteracting heat loss at high altitude.

## Introduction

High-altitude tourism and mountaineering is becoming ever more popular, especially on the world’s highest mountains in the Himalayas. More than 47,000 climbers have participated in expeditions to 8,000 m peaks in the Himalayas since the first ascents in 1950 until 2021, and 19,000 of them have reached 8,000 m summits (Salisbury and Hawley, 2020). Rarely did more than 100 climbers a year attempt Himalayan peaks above 6,000 m between 1950 and 1969. Thereafter the numbers increased gradually and more than 1500 climbers a year have been attempting these peaks in the twenty-first century (Salisbury and Hawley, 2007). Commercial climbing gained popularity in the early 1990s, when commercial expeditions contributed about 20% of all climbers attempting peaks over 6,000 meters; by 2006 climbers in commercial expeditions constituted almost 75% of attempts (Salisbury and Hawley, 2007). Two of the four most popular commercial climbing routes are on Mt. Everest and the number of climbers attempting this peak has risen 60% over the past 15 climbing seasons (Huey et al., 2020). Winter mountaineering is also gaining popularity (Benavides, 2021).

Knowledge of the Himalayan climate remains incomplete. Long-term weather stations do not exist and planners therefore lack extended observational series. Temporal meteorological observations are still made mostly during short climbing expeditions. The most important research on how Himalayan climate affects human physiology involves barometric pressure, air temperature and wind speed, the meteorological parameters that most limit human performance and survival at high altitudes. These parameters are derived from direct assessments at high altitude (West et al., 1983b; West, 1999), radiosonde data (West, 1996) and lately reanalysis data such as those from the US National Centers for Environmental Prediction (NCEP) (Kalnay et al., 1996; Moore and Semple, 2011) and the ERA5 from the European Centre for Medium-Range Weather Forecasts (Hersbach et al., 2020; Matthews et al., 2020a; Szymczak et al., 2021a, b). The state-of-the-art ERA5 data have a spatial resolution of 0.25° (about 28 km at the equator) at hourly intervals. Meteorological variables are interpolated from nearby stations and provide only an approximated picture of conditions on the mountain. A recent breakthrough project has provided detailed *in situ* values of many meteorological parameters with the installation of five automatic weather stations on the slopes of Everest by the National Geographic expedition in 2019 (Matthews et al., 2020a, b; National Geographic, 2021).

Barometric pressure determines the partial pressure of inspired oxygen (PiO_2_) that is critical for physiological performance, maximum oxygen uptake (VO_2_max), and the speed of vertical ascent at extreme altitude (West and Wagner, 1980; West et al., 1983a, 2007b; Bailey, 2001; Matthews et al., 2020a). Low air temperature and high wind speed mainly determine the risk of hypothermia and frostbite (Huey and Eguskitza, 2001). Firth et al. (2008) observed that severe weather is responsible for about 25% of fatalities above 7,000 m during ascents of Everest (Firth et al., 2008). Hypothermia is responsible for 16% of all deaths on Denali (McIntosh et al., 2008).

The levels and changes in barometric pressure and PiO_2_ at different high-altitude locations has been explored extensively (West et al., 1983b; West, 1996; Matthews et al., 2020a, b; Szymczak et al., 2021a, b). Precise calculations of barometric pressure and PiO_2_ at different altitudes have enabled analyses of how levels of hypobaric hypoxia affect humans in high-altitude expeditions (Grocott et al., 2010; Milledge, 2010; West, 2010) and in simulated conditions (Houston et al., 1987; Richalet et al., 1999).

Knowledge of the influence of hypobaric hypoxia on humans can be considered satisfactory, but hypothermic stress at high altitude has been studied only with simple parameters such as temperature, wind speed, wind chill temperature (WCT), and facial frostbite time (FFT) (Moore and Semple, 2011; Szymczak et al., 2021a, b). The standard equations for WCT (Osczevski and Bluestein, 2005) and FFT (Tikuisis and Osczevski, 2003) do not include variables that significantly determine high-altitude heat balance, such as solar radiation (Pugh, 1962) and air density (Huey et al., 2001). As air temperature, wind speed, WCT and FFT provide only general estimates of the hypothermic stress at high altitude, climbers need a more complete human heat balance analysis index, which would foster research on high-altitude thermal stress and enable more precise assessments of hypothermic stress (Szymczak et al., 2021a, b).

High-altitude climatic and physiological data that has recently become available enables more precise assessments of hypothermic stress by using models of human heat balance that include variables such as metabolic heat production, radiation balance, heat exchange by convection and conduction, and heat lost by evaporation and respiration. Our study thus aimed to analyze human heat balance at high altitudes during the active phase of climbing Mt. Everest in different weather conditions and seasons.

## Materials and Methods

### Materials

**FIGURE 1:**
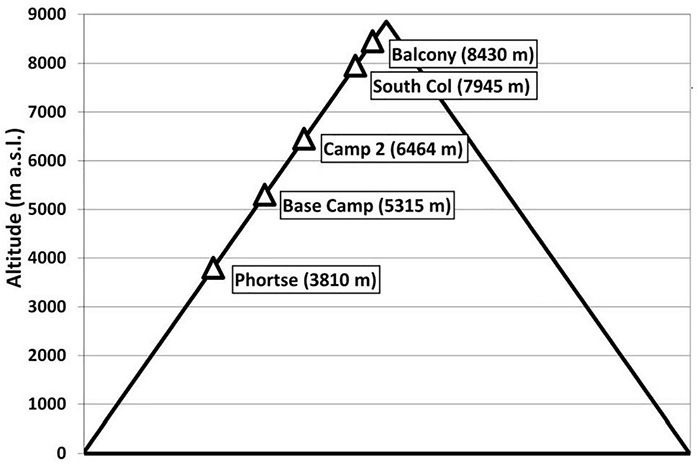
Altitude and location of meteorological stations on Mt. Everest’s Southeast Ridge Route installed during the National Geographic expedition.

We calculated heat balance characteristics of climbing Mt. Everest in different seasons using meteorological data from May 2019 to December 2020 collected by five automatic weather stations installed on the mountain between 3810 m (Phortse) and 8430 m (Balcony) (Figure 1) during a National Geographic expedition (Matthews et al., 2020a, b; National Geographic, 2021). The data included air temperature (Ta,°C), air vapor pressure (vp, hPa), relative air humidity (RH,%), mean wind speed (v, m⋅s^–1^), maximum wind speed (vmax, m⋅s^–1^), air pressure (ap, hPa), global solar radiation (Kglob, W⋅m^–2^), back (sky) longwave radiation (La, W⋅m^–2^), and outgoing ground longwave radiation (Lg, W⋅m^–2^) (Tables 1, 2). The data represent the hourly average values of the measured meteorological parameters and the maximum wind speed.

**TABLE 1:** List of abbreviations in alphabetical order.

ac, clothing albedo (%)
ap, air pressure (hPa)
BMR, basic metabolic rate (W⋅m^–2^)
C, convective heat exchange (W⋅m^–2^)
E, evaporative heat loss (W⋅m^–2^)
Epot, potential values of evaporative heat loss (W⋅m^–2^)
FFT, facial frostbite time (min)
h, height of the Sun (°)
hc, coefficient of heat transfer by convection (K⋅W^–1^⋅m^–2^)
hc’, coefficient of heat transfer by conduction within clothing (K⋅W^–1^⋅m^–2^)
he, coefficient of heat transfer by evaporation (hPa⋅W^–1^⋅m^–2^)
Icl, thermal insulation of clothing (clo)
Irc, coefficient reducing convective and radiative heat transfer due to clothing (dimensionless)
Ie, coefficient reducing evaporative heat transfer due to clothing (dimensionless)
Kglob, total global solar radiation (W⋅m^–2^)
Kt, global solar radiation of the cloudless sky (W⋅m^–2^)
L, net long-wave radiation (W⋅m^–2^)
La, reverse radiation of the atmosphere, back (sky) longwave radiation (W⋅m^–2^)
Lg, thermal radiation emitted by the surface, outgoing ground longwave radiation (W⋅m^–2^)
Ls, radiation emitted by the surface of the body/clothing (W⋅m^–2^)
M, metabolic heat production (W⋅m^–2^)
Mrt, mean radiant temperature (°C)
Ov, oxygen volume (g⋅m^–3^)
PiO_2_, partial pressure of inspired oxygen (hPa)
Q, radiation balance (W⋅m^–2^)
R, absorbed solar radiation (W⋅m^–2^)
Res, respiration heat loss (W⋅m^–2^)
RH, relative humidity of air (%)
S, heat transfer balance or changes in the body’s heat content (W⋅m^–2^)
SW, water loss due to sweating (g⋅h^–1^)
Ta, air temperature (°C)
Tsk, average skin temperature (°C)
w, degree of skin moisture (dimensionless)
WCT, wind chill temperature (°C)
v, mean wind speed (m⋅s^–1^)
v’, speed of movement (m⋅s^–1^)
vmax, maximum wind speed (m⋅s^–1^)
VO_2_max, maximum oxygen uptake (mlO_2_⋅kg^–1^⋅min^–1^)
vp, water vapor pressure in the ambient air (hPa)
vp’, vapor pressure equal to 5% of RH (hPa)
vps, water vapor pressure on the surface of the skin (hPa)

**TABLE 2:** Characteristics of the meteorological stations installed on Everest by the National Geographic expedition.

**Station**	**Latitude (°N)**	**Longitude (°E)**	**Elevation (m a.s.l.)**	**Period of observations start → end**	**Measured variables**
Phortse	27.8456	86.7472	3810	25 Apr 2019→ 31 Dec 2020	Ta, vp, RH, v, vmax, ap, Kglob, La, Lg
Base camp	27.9952	86.8406	5315	10 Oct 2019→ 31 Dec 2020	Ta, vp, RH, ap
Camp 2	27.9810	86.9023	6464	8 May 2019→ 31 Dec 2020	Ta, vp, RH, v, vmax, ap, Kglob, La, Lg
South col	27.9719	86.9295	7945	21 May 2019→ 31 Dec 2020	Ta, vp, RH, v*, vmax*, ap, Kglob, La, Lg
Balcony	27.9826	86.9292	8430	22 May 2019→ 20 Jan 2020	Ta, vp, RH^#^, v^&^, vmax^&^, ap

*Ta, air temperature; vp, air vapor pressure; RH, relative air humidity; v, mean wind speed; vmax, maximum wind speed; ap, air pressure; Kglob, global solar radiation; La, back (sky) longwave radiation; Lg, outgoing ground longwave radiation.*

*Data questionable after 6 Jan 2020; ^#^data available till 20 Dec 2019; ^&^data available till 24 Oct 2019.

The stations started and finished their measurements on different dates and recorded different variables (Table 2). The longest and most complete series of data were provided by stations in Phortse, Camp 2 and South Col. Fewer parameters were measured at Base Camp and at the highest station of Balcony, where the measurements ended on 20 January 2020. Balcony’s wind speed measurements became unreliable after 25 October 2019 because of the extreme conditions and the anemometer barely indicated air movement from mid-December 2019. The station’s air humidity sensor malfunctioned on 20 December 2019. Wind speeds recorded at the South Col station after 6 January 2020 are also doubted (Matthews et al., 2020b; Table 2).

Meteorological conditions clearly differed at the stations between May 2019 and May 2020 based on their altitudes. Mean air pressure ranged from about 355 hPa at the highest station (Balcony) to 646 hPa at the lowest (Phortse). Mean air temperature ranged from –23°C at Balcony to 4°C at Phortse. Temperature extremes also ranged widely. The highest temperature (17°C) was registered at Phortse and the lowest (–45°C) at Balcony. Global solar radiation increased with altitude, with the highest momentary values ranging from 1,306 W⋅m^–2^ at Phortse to 1,692 W⋅m^–2^ at South Col. Wind speed was highest at the highest stations (South Col and Balcony). The highest mean hourly values of wind speed reached 26 m⋅s^–1^ at the exposed South Col site, which also recorded the most extreme wind gusts of about 44 m⋅s^–1^.

Large seasonal variations were recorded for air temperature, solar radiation and wind speed. The air temperature ranged over 15°C at Phortse from the warmest in August 2019 to the coldest in January 2020. The annual range at the South Col station was 19°C. Clear seasonal differences were observed in the total daily sum of global solar radiation, with the widest range of about 30 MJ at South Col between May and December 2019. Seasonal differences were smaller at other stations, ranging over about 20 MJ at Camp 2 and over 10 MJ at Phortse. Large seasonal changes in wind speed were recorded at the elevated stations: South Col showed the greatest variability in wind speed (*SD* = 6 m⋅s^–1^) and maximum wind speed (*SD* = 10 m⋅s^–1^) (Table 3).

**TABLE 3:** Meteorological parameters at Mt. Everest stations between 1 May 2019 and 31 May 2020: means ± standard deviation (SD), (minimum; maximum values).

**Station**	**Air pressure (hPa)**	**Air temperature (°C)**	**Air vapor pressure (hPa)**	**Relative humidity (%)**	**Global solar radiation (W⋅m^–2^)**	**Wind speed (hourly mean, m⋅s^–1^)**	**Wind gust (m⋅s^–1^)**
Phortse	646 ± 3 (637; 654)	4 ± 5 (–12; 17)	7.0 ± 3 (1; 13)	78 ± 17 (7; 78)	216 ± 68 (0; 1306)	1 ± 0 (0; 6)	3 ± 1 (–; 13)
Base Camp*	531 ± 3 (520; 540)	–7 ± 4 (–21; 6)	1.8 ± 1 (0.1; 6)	45 ± 27 (3; 45)	–	–	–
Camp II	460 ± 4 (446; 468)	–11 ± 6 (–31; 4)	1.7 ± 1 (0.1; 5)	48 ± 27 (4; 48)	249 ± 92 (0; 1527)	3 ± 3 (0; 23)	7 ± 5 (–; 35)
South Col	377 ± 6 (358; 387)	–22 ± 8 (–40; –1)	0.8 ± 1 (0.1; –3)	52 ± 24 (3; 52)	334 ± 139 (0; 1692)	9^&^ ± 6 (0; 26)	16^&^ ± 10 (–; 44)
Balcony**	355 ± 6 (334, 362)	–23 ± 9 (–45; –1)	1.0 ± 1 (0.1; 2)	71 ± 13 (5; 70)	–	7^#^ ± 2 (0; 19)	8^#^ ± 4 (–; 34)

*10th Oct 2019–31st May 2020; ** 22nd May 2019–17th Jan 2020; ^#^22nd May–24th Oct 2019; ^&^22nd May 2019–6th Jan 2020.

Given the doubts about the accuracy of some observational data, mainly wind speed, and the large seasonal differences measured in most of the weather parameters, we selected data from three stations (Phortse, Camp 2, South Col) over periods representing summer and winter for our detailed analysis of heat balance and bio-meteorological indicators. We selected 1 May to 15 August 2019 to represent the summer season (Camp 2 from 8 May, South Col from 21 May) and 16 November 2019 to 6 January 2020 for the winter season. These periods correspond with the climbing and winter seasons in the Himalayas and Karakoram, so the dates have practical significance for climbers.

Significant intraseasonal variation of radiation and wind led to complex permutations of meteorological data and human heat balance, so we divided summer and winter season data into four broad weather ranges to improve the utility of our analysis: (1) cloudy and weak wind, (2) cloudy and strong wind, (3) sunny and weak wind, (4) sunny and strong wind.

Hourly average values of wind speed and solar radiation from the National Geographic weather stations (National Geographic, 2021) were averaged for each day and then season (summer, winter) for each station. Mean values of wind speed and global solar radiation calculated individually for each season and each station were adopted as thresholds separating the categories of wind speed (weak or strong) and solar radiation (cloudy or sunny). Daily values below the thresholds were categorized as weak (for wind speed) and cloudy (for solar radiation); those above the thresholds were categorized as strong and sunny. Each day at each station was then assigned into one of the four weather groups.

We averaged hourly values of the meteorological variables (v, Kglob, ap, Ta, vp, and RH) for the day and then for four weather categories for each station in each season (Table 4), then used these values in our calculations of the components of heat balance. In most cases these values differed significantly at *p* = 95%. At Phortse and Camp 2, weak and strong wind speed did not differ between cloudy and sunny days, in summer and in winter.

**TABLE 4:** Mean values of meteorological variables in particular weather categories at different stations and seasons during summer (1 May–15 August) and winter (16 October–6 January) seasons.

			**Wind speed**		**Global solar**		**Air pressure**		**Air temperature**		**Relative**		**Air vapor**	
			**(m⋅s^–1^)**		**radiation (MJ⋅day^–1^)**		**(hPa)**		**(°C)**		**humidity (%)**		**pressure (hPa)**	
**Station**	**Season**	**Weather type**	**Weak wind**	**Strong wind**	**Weak wind**	**Strong wind**	**Weak wind**	**Strong wind**	**Weak wind**	**Strong wind**	**Weak wind**	**Strong wind**	**Weak wind**	**Strong wind**
Phortse	Summer	Cloudy	1.1	1.5	15.1	16.6	645.8	645.3	9.5	6.8	94.7	89.8	11.2	9.9
		Sunny	1.2	1.7	23.1	25.8	646.7	646.5	10.7	8.3	93.0	80.6	11.9	8.9
	Winter	Cloudy	0.9	1.5	9.6	12.5	646.3	644.4	–3.6	–3.2	76.9	58.4	4.8	3.1
		Sunny	1.0	1.4	17.3	17.3	646.0	647.8	–2.2	–1.8	64.2	48.5	4.1	3.6
Camp 2	Summer	Cloudy	0.9	2.1	23.8	19.9	463.3	462.6	–3.8	–8.6	85.6	59.5	4.1	2.0
		Sunny	1.0	1.9	30.9	32.3	464.0	462.6	–3.5	–6.4	77.7	45.2	3.8	1.8
	Winter	Cloudy	3.5	8.5	11.6	9.1	458.3	455.4	–17.6	–19.6	30.3	32.0	0.6	0.5
		Sunny	3.5	8.8	16.9	14.6	461.1	458.3	–16.6	–17.6	21.3	15.0	0.5	0. 3
South Col	Summer	Cloudy	2.3	8.5	29.4	40.2	383.8	382.2	–10.9	–14.3	88.1	57.1	2.4	1.2
		Sunny	2.9	7.8	44.4	46.5	383.6	381.6	–11.5	–15.4	72.6	52.2	1.9	1.0
	Winter	Cloudy	3.9	15.6	9.2	10.4	370.5	374.7	–33.5	–30.2	60.4	65.6	0.3	0.5
		Sunny	3.2	14.9	17.3	18.3	370.9	375.9	–34.0	–28.4	27.7	32.2	0.2	0.3

### Methods

We used the Man-Environment Heat Exchange Model (MENEX-2005) (Błażejczyk, 1994, 2005a,b; Błażejczyk et al., 2012) to calculate the components of human heat balance. The model is sensitive to changes in basic meteorological elements, but unlike models such as the Universal Thermal Climate Index (UTCI) Fiala model (Fiala et al., 2012), the Munich Energy-balance Model for Individuals (MEMI) (Hoppe, 1993) and the Klima-Michel-Model (KMM) (Jendritzky et al., 1990), MENEX-2005 accounts for the physical parameters of air that characterize the high-altitude environment: air density and air oxygen content at different altitudes above sea level (Błażejczyk et al., 2012). The model can also be used in different bioclimatic applications such as recreation, tourism, climatotherapy, health prophylaxis, and urban climatology, or in thermophysiological applications such as working conditions and sports physiology.

The MENEX_2005 model calculates the basic components of heat balance under given environmental conditions in non-stationary conditions. The method of calculating individual components of human heat balance are described by Błażejczyk and Matzarakis (2007) and Błażejczyk and Kunert (2011) and in our Annexure.

**FIGURE 2:**
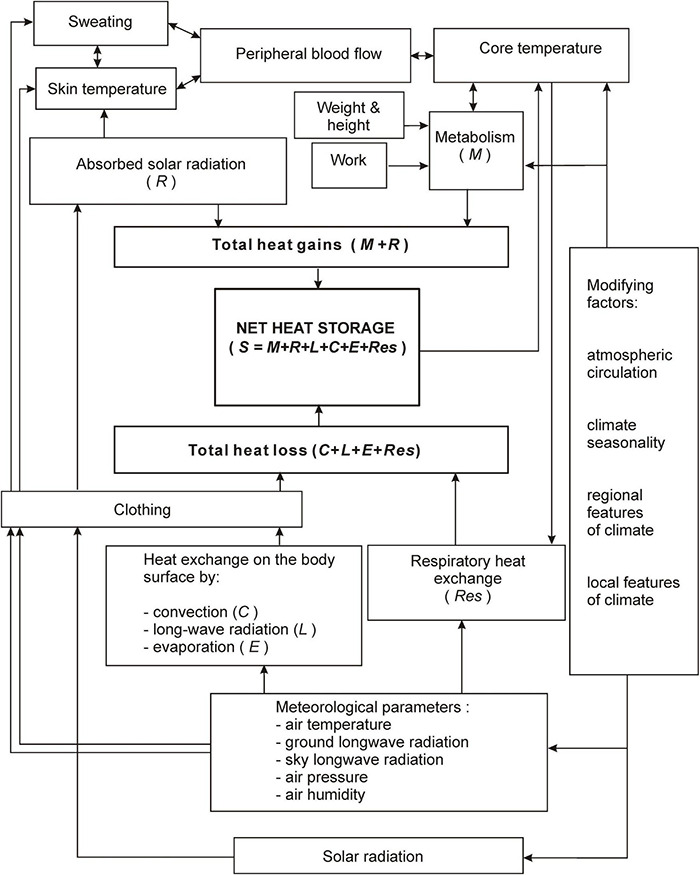
Relationships between meteorological and physiological factors of Man-Environment Heat Exchange Model (MENEX-2005). Adapted from Błażejczyk (1994).

The general equation of heat transfer between humans and the environment used in the MENEX_2005 model is:


(1) M+Q+C+E+Res=S

where: M is metabolic heat production, including basic metabolic rate (BMR) and metabolism related to physical activity; Q is radiation balance in humans; C is convective heat exchange; E is evaporative heat loss; Res is respiration heat loss; S is heat transfer balance or changes in the body’s heat content. Radiation balance in humans is the sum of absorbed solar radiation (R) and net long-wave radiation (L) (Figure 2). All heat fluxes are expressed in W⋅m^–2^. The model will not account for heat losses to conduction because of the low values of this heat flux in a moving, upright human.

The model’s inputs include meteorological and physiological variables. The meteorological information it requires include air temperature (Ta,°C), wind speed (v, m⋅s^–1^), water vapor pressure (vp, hPa), relative air humidity (RH,%), atmospheric pressure (ap, hPa) and total solar radiation (Kglob, W⋅m^–2^). Meteorological parameters are estimated at the height of a standing person’s torso, about 1.2 m above ground level (Jendritzky et al., 2012). The physiological data the model requires include metabolic heat production (M, W⋅m^–2^), thermal insulation of clothing (Icl, clo), clothing albedo (ac,%), speed of movement (v’, m⋅s^–1^), average skin temperature (Tsk,°C) and skin moisture content (w, without dimension). Physiological parameters such as average skin temperature and skin moisture content were calculated using empirical formulas (Annexure). We used values for these variables that were appropriate to the conditions we analyzed: metabolic heat production 190 and 290 W⋅m^–2^; clothing insulation of 3.5, 4.5, and 5.5 clo; albedo equal to 30; and an average speed of movement while climbing of 0.05 m⋅s^–1^.

The daily energy expenditure of climbers on high-altitude expeditions depends on altitude. Energy expenditure averages about 14.7 MJ in climbs between 2,500 and 4,800 m (Westerterp et al., 1992), and ranges from 13.6 to 20.6 MJ on Himalayan 8,000 m peak expeditions while climbing above 5,000 m (Pulfrey and Jones, 1985; Westerterp et al., 1992; Reynolds et al., 1999; West et al., 2007c). The steeper slopes that climbers encounter at higher elevations, which require more effort, might partially explain the higher energy expenditures at these altitudes (Watts et al., 1999; Bertuzzi et al., 2007; Ainsworth et al., 2011; Sas-Nowosielski and Wycislik, 2019). BMR is the largest component of daily energy expenditure at high altitude because periods of intense activity seldom last long (West et al., 2007c). Based on the experience of climbers in the Himalayas, we assumed that in a typical day climbers spend 8 h climbing and 16 h resting and sleeping. BMR of acclimatized alpinists at 5,800 m is 10% higher than at sea level (Gill and Pugh, 1964). We estimated climbers’ daily energy expenditure at 15 MJ for locations at or below the Everest base camp (Phortse—3810 m, Base Camp—5,315 m) and 20 MJ for higher locations (Camp 2—6,464 m and South Col—7,945 m). Given these assumed daily energy expenditures, the characteristics of a typical climbing day, and a 10% increase of BMR at altitude, we calculated the values of metabolic heat production during active climbing to be 3 METs (≈190 W⋅m^–2^) for lower altitudes and 5 METs (≈290 W⋅m^–2^) for higher altitudes.

The thermal insulation of clothing used when climbing in the Himalayas was adopted on the basis of ISO 9920 (2007) and ISO 11079 (2007), Havenith’s research (2010) and research conducted during the creation of the UTCI index (Havenith et al., 2012). The clothing insulation values we adopted were verified by IREQ model (Holmér, 1998) and information from participants in Himalayan expeditions organized by Polish Mountaineering Association. Clothing with 3.5 clo insulation is suitable mainly for the summer season in areas with relatively high air temperature, so we assumed it was enough to sustain thermal balance while climbing lower areas of Everest: Phortse (summer and winter) and Camp 2 (summer). Thick clothing with 4.5 clo insulation is mainly used in winter and when air temperatures drop below –15°C, as at Camp 2 in winter. Clothing with a thermal insulation of 5.5 clo is used in the winter season while climbing in the sub-peak zone of Mt. Everest, such as South Col, because of the extremely low temperatures and high wind speeds.

We determined human heat balance at different altitudes by using the values of heat transfer fluxes (convective, evaporative and respiration heat losses, absorbed solar radiation, and net long-wave radiation) and the water lost to sweating. We based our calculations of these variables on mean daily values of meteorological parameters that we calculated for each station, season, and weather category.

The characteristics of the human heat balance were complemented by oxygen volume (Ov, g⋅m^–3^). Oxygen volume determines the weight of oxygen in the air, which depends on air temperature, water vapor pressure, and atmospheric pressure, which changes with altitude above sea level. Oxygen volume is a bio-meteorological indicator used to assess the load on the respiratory system (Wojtach, 2003; Lecha Estela, 2018). Oxygen volume was calculated using the Błażejczyk and Kunert’s (2011) equation.


(2) Ov=[80.51⋅ap/(Ta+273)]⋅(1-vp/ap)

### Statistical Analysis

The statistical significance of the different heat balance characteristics calculated for each station, season and type of weather was verified with Stragraphics Centurion XVI, version 16.2.04, at the 95% confidence level.

## Results

### General Characteristics of Human Heat Balance Components

Given the average values of the characteristic human heat balance for the observation period (1 May 2019 to 6 January 2020) that we calculated for various combinations of metabolic heat production and clothing insulation, the largest statistically significant differences between the stations involved convective heat loss, which increased with altitude. At the highest station of South Col, convective heat loss was about 4–5 times larger than at Phortse. Net long-wave radiation and respiration heat loss also increased with altitude. The flux of absorbed solar radiation increased with altitude and had a positive value, causing heat gain. Little heat was absorbed in this way, however.

Altitude had no significant effect on the intensity of evaporative heat loss and the amount of water lost to sweating: these variables depended on the level of physical activity. With a metabolic heat production of 290 W⋅m^–2^ they were significantly 1.5–2 times greater than at 190 W⋅m^–2^. Increased physical activity also resulted in more respiration heat loss. Physical activity had little effect on the amount of heat lost to convection, net long-wave radiation or absorbed solar radiation: these variables depended on the degree of clothing insulation. Clothing with an insulation of 4.5 clo was significantly more effective in protecting the body against losses to convection and long-wave radiation, but also significantly reduced the amount of absorbed solar radiation (Table 5).

**TABLE 5:** Mean values of human heat balance variables at different stations 1 May 2019 to 6 January 2020 for different combinations of metabolic heat production and clothing insulation.

**Metabolic heat production**	**Heat balance variables**	**Phortse**	**Camp 2**	**South Col**	**Phortse**	**Camp 2**	**South Col**
			
		**Icl = 3.5 clo**			**Icl = 4.5 clo**		
M = 190 (W⋅m^–2^)	C (W⋅m^–2^)	–20	–32	–92	–16	–26	–77
	E (W⋅m^–2^)	–53	–51	–51	–53	–51	–51
	L (W⋅m^–2^)	–13	–18	–29	–11	–14	–24
	Res (W⋅m^–2^)	–24	–29	–33	–24	–29	–33
	R (W⋅m^–2^)	4	5	10	4	4	8
	SW (g/hour)	141	134	133	141	134	133
M = 290 (W⋅m^–2^)	C (W⋅m^–2^)	–20	–32	–92	–16	–26	–77
	E (W⋅m^–2^)	–96	–94	–93	–96	–94	–93
	L (W⋅m^–2^)	–13	–18	–30	–11	–14	–25
	Res (W⋅m^–2^)	–37	–45	–50	–37	–45	–50
	R (W⋅m^–2^)	4	5	11	4	4	9
	SW (g/hour)	251	243	242	251	243	242

The structure of heat loss fluxes changed with increased altitude. Heat lost to evaporation clearly dominated at the altitude of Phortse and Camp 2, where they reached 40–60%, depending on clothing insulation and metabolic heat production. At the highest station of South Col the convection flux contributed significantly more. Convection accounted for 41% of heat loss at a clothing insulation of 3.5 clo with metabolic heat production of 190 W⋅m^–2^. With increased effort (M = 290 W⋅m^–2^) and with thicker insulation (4.5 clo), convective heat loss at South Col (31%) was similar to evaporative heat loss (38%) (Table 5).

### Components of Heat Balance

The components of heat balance with the clothing insulation and physical activity we assumed were clearly differentiated by the altitude above sea level, the season and the type of weather. When considering days with different weather types in summer (1 May-15 August 2019) and winter (16 October 2019 to 6 January 2020) the greatest variation occurs in convection. Convection ranged from –16 W⋅m^–2^ at Phortse in summer with cloudy and weak wind to –182 W⋅m^–2^ at South Col in winter with cloudy and strong wind. Net long-wave radiation in humans ranged from about –15 W⋅m^–2^ at Phortse in both seasons and in all-weather categories to –38 W⋅m^–2^ at South Col in winter with cloudy and strong wind. Absorbed solar radiation values ranged from 2.8 W⋅m^–2^ at Phortse in summer with cloudy and weak wind to 11.1 W⋅m^2^ at South Col in summer with sunny and strong wind. The evaporative heat loss differed clearly between Phortse at about –53 W⋅m^–2^ and Camp 2 and South Col at about –93 W⋅m^–2^. These large differences resulted from the metabolism values we adopted of 190 W⋅m^–2^ for Phortse and 290 W⋅m^–2^ for Camp 2 and South Col. The amount of water lost by sweating, which ranged from 135 to 144 g⋅h^–1^ at Phortse to 240–243 g⋅h^–1^ at Camp 2 and South Col resulted from evaporative heat loss and metabolic heat production (Table 5).

The contrasts within components of heat flux in the weather categories we compared increased with higher altitude. For example, convection was higher during strong wind days at Phortse in winter by about 11% on sunny days to 16% on cloudy days than on winter days with weak wind. Convection on windy days at South Col in winter was as much as 4.6 times greater on cloudy days to 4.8 times greater on sunny days than on weak wind days. Contrasts in absorbed solar radiation increased with altitude on sunny and cloudy days. At Phortse the seasonal difference in absorbed solar radiation in summer was 1.5 (weak wind) to 1.6 (strong wind) times greater on sunny days than on cloudy days, and in winter 1.5 (strong wind) to 1.95 (weak wind) times greater on sunny days. At South Col the relationship of absorbed solar radiation on sunny and cloudy days ranged from 1.2 (strong wind) to 1.7 (weak wind) times greater on sunny summer days and 2.2 (strong wind) to 2.6 (weak wind) times greater on sunny winter days than on cloudy days.

### Total Values and Structure of Heat Loss Fluxes

**FIGURE 3:**
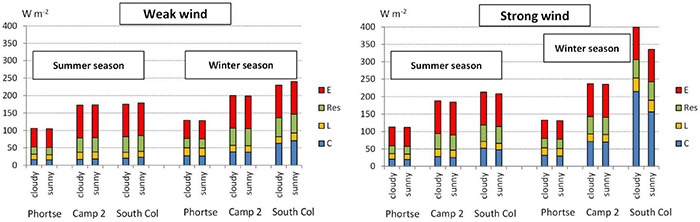
Totals and structure of heat loss fluxes in different seasons and weather categories at particular stations. C, convective heat loss; L, net long-wave radiation in humans; Res, respiration heat loss; E, evaporative heat loss.

The level of heat loss with the clothing insulation values we selected and physical activity clearly differed with season and altitude. Wind and radiation conditions played an important role with greater heat loss in winter than in summer. At the highest station of South Col heat loss reached 400 W⋅m^–2^ in winter in cloudy and strong wind days; in summer heat loss ranged from 180 to 215 W⋅m^–2^. At the lowest point, Phortse, total heat loss varied from about 110 W⋅m^–2^ in summer to about 130 W⋅m^–2^ in winter, depending on wind and radiation (Figure 3).

### Net Heat Storage

The effect of heat exchange between the human body and the environment was a heat storage value that indicated whether heat was accumulated in the body or was eliminated, leading to the body cooling. In general, climbing caused as much as 124 W⋅m^–2^ of heat to accumulate in the human body, which might led to overheating as an effect of intensive activity with much internal heat production linked to the high thermo-insulating properties of mountaineering clothing. Only at the highest parts of Mt. Everest in winter were alpinists risk of overcooling, even while climbing intensively in heavy clothing (Table 6). Our calculations showed that alpinists faced little risk of overheating or overcooling while actively climbing. Our calculations consider only alpinists as they undergo physical exertion; the heat balance of climbers who rest in a tent or are forced to bivouac—unplanned camping, usually without a tent, because of bad weather or ill health—needs further scientific exploration.

**TABLE 6:** Average values of heat balance components and net heat storage of climbers in different weather scenarios at particular stations during summer (1 May–15 August) and winter (16 October–6 January) seasons.

			**Convection**		**Long-wave**		**Absorbed solar**		**Respiration**		**Evaporation**		**Sweating**		**S – net heat**	
			**(W⋅m^–2^)**		**radiation (W⋅m^–2^)**		**radiation (W⋅m^–2^)**		**(W⋅m^–2^)**		**(W⋅m^–2^)**		**(g⋅h^–1^)**		**storage (W⋅m^–2^)**	
**Station (M) (W⋅m^–2^)**	**Season (Icl) (clo)**	**Weather category**	**Weak wind**	**Strong wind**	**Weak wind**	**Strong wind**	**Weak wind**	**Strong wind**	**Weak wind**	**Strong wind**	**Weak wind**	**Strong wind**	**Weak wind**	**Strong wind**	**Weak wind**	**Strong wind**
Phortse M = 190	Summer Icl = 3.5	Cloudy	–16	–21	–15	–16	3	3	–22	–23	–53	–53	142	141	**87**	**80**
		Sunny	–15	–20	–15	–16	4	5	–21	–23	–54	–54	144	144	**90**	**83**
	Winter Icl = 3.5	Cloudy	–27	–31	–23	–22	3	4	–28	–28	–52	–52	135	137	**64**	**61**
		Sunny	–26	–29	–23	–22	6	7	–27	–27	–52	–53	137	138	**68**	**65**
Camp 2 M = 290	Summer Icl = 3.5	Cloudy	–17	–28	–20	–22	5	4	–42	–45	–93	–93	243	243	**122**	**106**
		Sunny	–18	–26	–20	–21	7	7	–42	–44	–94	–94	244	244	**124**	**113**
	Winter Icl = 4.5	Cloudy	–38	–72	–19	–22	3	3	–49	–50	–93	–93	242	242	**93**	**56**
		Sunny	–37	–70	–19	–21	5	5	–49	–50	–93	–94	242	243	**96**	**60**
South Col M = 290	Summer Icl = 4.5	Cloudy	–20	–52	–17	–20	6	10	–46	–48	–93	–94	242	243	**120**	**87**
		Sunny	–23	–48	–17	–19	9	11	–46	–48	–93	–93	242	243	**120**	**93**
	Winter Icl = 5.5	Cloudy	–40	–182	–25	–38	3	4	–56	–55	–93	–93	240	241	**63**	**–105**
		Sunny	–32	–152	–27	–34	7	9	–56	–54	–92	–93	240	241	**56**	**–35**

### Effects of Air Pressure and Solar Radiation on Elements of Heat Flux

**FIGURE 4:**
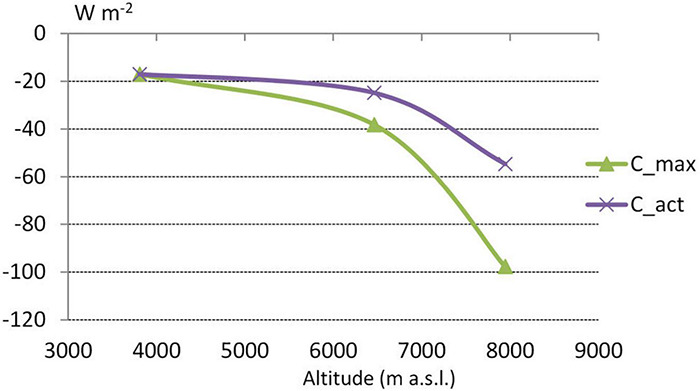
The dependence of the convective heat loss (C) value on the level of atmospheric pressure at different stations 1 May 2019 to 6 January 2020 for metabolic heat production of 290 W⋅m^– 2^ and clothing insulation 5.5 clo. C_act—actual C values observed at particular stations, C_max—potential C values from air pressure recorded at Phortse station.

The differences we observed in the characteristics of the heat balance at each station resulted from the complex interaction of meteorological elements. The altitude-related decrease in atmospheric pressure and air density, along with the increased solar radiation at height, significantly affected the values that we calculated for convective heat loss and absorbed solar radiation. For example, with Icl = 5.5 clo and M = 290 W⋅m^–2^, the average value of convective heat loss at South Col in the period between 1 May 2019 and 6 January 2020 (ap = 380 hPa, Ta = –18.9°C and v = 8.8 m⋅s^–1)^ was about 54 W⋅m^–2^ (Table 5). At Phortse (ap = 650 hPa), the convection heat loss would be 1.8 times greater with the same temperature and wind speed. The values of convective heat loss observed at higher stations (Camp 2, South Col) were smaller than if the atmospheric pressure were the same as at Phortse had the air temperature and wind speed been unchanged. At altitudes between 6,500 and 8,000 m convective heat losses are relatively lower than at an altitude of 3,800 m (Figure 4).

As with convective heat loss, absorbed solar radiation also increased with height: at the highest part of Mt. Everest it was 1.7 times the comparable value at Phortse.

### Oxygen Volume

An important problem of a sojourn at high altitude is the amount of oxygen in the air, which decreases with altitude. Oxygen volume is 277 g⋅m^–3^ under the reference conditions of 1,000 hPa, a temperature of 15°C and water vapor pressure of 8 hPa. At the stations we analyzed on the route up Mt. Everest mean oxygen volume values decreased from 186 g⋅m^–3^ at Phortse to 141 g⋅m^–3^ at Camp 2 and 121 g⋅m^–3^ at South Col. These values represented 67, 51, and 44% of the values determined under the reference conditions. Climbers compensate for the reduced amount of oxygen in the air by breathing more intensely, which became evident when we compared the amount of heat lost to respiration under different metabolic conditions. At a metabolic rate of 290 W⋅m^–2^ we found that 1.6 times more heat was lost to respiration under the same thermal and humidity conditions than at M = 190 W⋅m^–2^ (Table 6). This marked increase in respiration heat loss resulted from greater exertion and therefore a higher breathing rate (Annexure, formula 23).

Oxygen volumes varied considerably between seasons and in different weather. In summer they were slightly higher than in winter, on cloudy days they were higher than on sunny days, and in strong winds they were higher than in light winds (Table 7). These differences were mainly caused by differences in air temperature (Table 4).

**TABLE 7:** Average values of oxygen volume in different weather scenarios at particular stations during summer (1 May–15 August) and winter (16 October–6 January) seasons.

			**Oxygen volume (g⋅m^–3^)**	
	**Season**	**Weather Category**	**Weak wind**	**Strong wind**
**Phortse**	Winter	Cloudy	181	183
		sunny	180	183
	Summer	Cloudy	191	191
		Sunny	190	191
**Camp 2**	Winter	Cloudy	137	140
		Sunny	138	139
	Summer	Cloudy	144	144
		Sunny	143	143
**South Col**	Winter	Cloudy	117	119
		Sunny	118	119
	Summer	Cloudy	124	123
		Sunny	122	123

## Discussion

### Meteorological Conditions

Throughout the troposphere the higher the altitude and the colder the season, the lower are barometric pressure and air temperature at a given latitude (Brunt, 1952; West, 1996; Whiteman, 2000). Global solar radiation increases as elevation increases because of the reduced optical atmospheric mass at altitude, where diffused solar radiation therefore increases. Solar radiation is also reflected from the ice and snow covering the higher south slopes around the measuring stations as reported for Everest at South Col (Matthews et al., 2020b) and for Tatry Mountains (Błażejczyk et al., 2013). The large seasonal differences that we found in wind speed at higher stations, with much higher winds in winter, accords with the seasonal trend in the global jet streams. The Subtropical component of the Northern Hemisphere jet stream flows between 20°N and 35°N, above the Himalayas in winter; this jet stream weakens in summer and shifts northwards (Archer and Caldeira, 2008; Pena-Ortiz et al., 2013).

We observed the most severe weather conditions that adversely affect human heat balance—low air temperature, high wind speed, and low solar radiation— at the highest station in winter. Alpinists should expect the lowest air temperatures, the lowest daily global solar radiation, and the strongest wind at the highest altitudes in winter. Those parameters of weather not only fall with altitude but their seasonal amplitudes also increase with altitude (National Geographic, 2021).

### Convective Heat Loss

Alpinists must contend with a large increase in convective heat loss with rising altitude as they climb Mt. Everest. Convective loss is 4–5 times higher at 8,000 m than at 4,000 m, which can be explained by the significant drop in air temperature and the higher wind speeds at higher altitudes. Convective heat loss is the parameter closely linked to altitude, season, and weather. Our results accord with the findings of other authors, who have calculated the lowest values of WCT and FFT on Everest’s summit in winter (Moore and Semple, 2011; Szymczak et al., 2021b). Climbers should also be aware that convective heat loss changes with the different weather categories they experience, especially in winter, along with altitude. At South Col in winter convective heat loss is almost 5 times higher in strong wind than in weak wind. This shows how abrupt changes in the weather can significantly affect human survival, as other authors have noted. Moore and Semple (2012) suggested that higher hypoxic and hypothermic stress due to weather changes is often responsible for climbers’ deaths (Moore and Semple, 2012). They presented the cases of two climbers who had to bivouac above 8,500 m on descent from Everest in extreme conditions: the first experienced air pressure of 333 hPa, air temperature of –31°C and wind speed of 15 m⋅s^–1^; the second 338 hPa, –23°C, and 2 m⋅s^–1^. The first climber died but the second survived. Moore and Semple (2012) attributed his death to the higher hypoxic and hypothermic stress he underwent.

### Convective Heat Loss and Air Pressure

Air density decreases at high altitudes because of the lower air pressure. Lower density provides better insulation in the near-body air layer, which reduces convective heat loss (Kandjov, 1997), as we observed in our results. With lower air pressure at higher altitudes convective heat loss will be lower than at lower altitudes with the same air temperature and wind speed. As presented in our results, at 8,000 m the lower air pressure reduced convective heat loss by almost 50% compared with convective loss at 4,000 m in the same air pressure and temperature. Convective heat loss that increases with altitude because of decreasing air temperature and increasing wind speed is partly ameliorated by the lower barometric pressure encountered at height. Our results concur with Huey et al. (2001) who calculated that with the 60% decline of air density from sea level to 9,000 m the convective heat loss at 9,000 m decreases by about 45% compared with the loss at sea level in the same conditions at a temperature of –33.5°C and wind speeds up to 28 m⋅s^–1^. The standard equations for WCT and FFT (Tikuisis and Osczevski, 2003; Osczevski and Bluestein, 2005) applied in other studies that analyzed hypothermic stress at high altitude (Moore and Semple, 2011; Szymczak et al., 2021a, b) presume sea-level densities of air and therefore significantly overestimate heat loss at altitude. Climbers usually use wind speed to identify suitable climbing weather windows (Peplow, 2004), but should therefore also consider the relationship between convective heat loss and air pressure to interpret weather forecasts at high altitude. The same wind speed causes less convective heat loss at 8,000 m than at 4,000 m.

### Clothing Insulation

Clothing insulation is one of the few easily controllable parameters that enable alpinists to limit convective heat loss and to maintain thermal equilibrium (Tikuisis, 1995; Havenith et al., 2012). Proper clothing insulation determines survival time in low air temperatures and high winds. Tikuisis (1995) used a mathematical model to predict survival times under sedentary conditions. These calculations determined that in environmental conditions similar to those at the South Col on an average winter day (air temperature –20°C, wind speed 14 m⋅s^–1^) a climber wearing one loose layer 1 mm thick would survive 3 h and a climber wearing three loose 1 mm layers of clothing would survive 12 h (Tikuisis, 1995).

Our calculations determined that clothing insulation of 4.5 clo would enable a climber to counteract the potential heat loss due to environmental conditions at all the stations, in all seasons, and in all-weather apart from the strong wind on South Col in winter (air temperature ∼ –30°C and wind speed of ∼ 15 m⋅s^–1^). In cloudy, windy, winter days at South Col we derived a net heat balance value of ∼ –70 W⋅m^–2^. Our results are similar to those published by Havenith (2010). He calculated the clothing insulation that a climber required in different air temperature and wind conditions for thermal equilibrium in a summit bid on Everest. The energy production during climbing the summit of Everest was assumed as 5 METs (Havenith, 2010). He calculated that clothing insulation of 3.5 clo was enough to maintain a climber’s thermal equilibrium in conditions from an air temperature of –30°C at a low wind speed of 2 m⋅s^–1^ to an air temperature of –10°C with high wind speed of 11 m⋅s^–1^. The environmental conditions that could be balanced with clothing insulation of 4.5 clo lay between Ta –40°C with v 2 m⋅s^–1^ and Ta –20°C with v 11 m⋅s^–1^, and for 5.5 clo a range between Ta < –45°C with v 2 m⋅s^–1^ and Ta –30°C with v 11 m⋅s^–1^ (Havenith, 2010). Considering these calculations, clothing insulation of 4.5 clo should maintain a climber’s thermal balance throughout ascents of Everest in the normal season (May, October), but 5.5 clo would be required for winter ascents (Szymczak et al., 2021a). Modern mountaineering clothing for extreme altitudes can provide insulation of about 5.6 clo (Havenith, 2010). Given the importance of clothing insulation to maintain thermal equilibrium and thus ensure survival, climbers should precisely calculate the insulation properties not only of their climbing outfits but also of their emergency survival bags and the shelters they need for bivouacs. Emergency insulation and protection from wind with a survival bag or shelter might help alpinists survive unforeseen situations. It should be taken into account that the very high insulation properties are needed to conserve thermal equilibrium at rest. Cena and Tapsell (2000) and Cena et al. (2003) observed that the insulation of clothes together with a sleeping bag needed to keep a climber’s thermal sensation in tent between neutral and slightly warm at 5,000 m might be as high as 7 clo. Emergency equipment should be chosen based on research for emergency medicine (Oliver et al., 2016; Haverkamp et al., 2018), nevertheless, this field needs further development, which would likely include the use of external chemical or electrical heaters.

Proper clothing insulation not only significantly decreases convective heat loss but also the heat loss to long-wave radiation. The reduction of heat lost to long-wave radiation is minor when compared to convection.

### Physical Activity Level

Levels of physical activity play an important role in human heat balance (Bligh and Johnson, 1973). Greater physical activity generates more metabolic heat, but also provokes heat loss to evaporation and respiration. We observed that when metabolic heat rises so does the net heat value despite the simultaneous increase in evaporation and respiration heat losses. When the metabolic heat production was increased by 100 W⋅m^–2^ from 190 to 290 W⋅m^–2^ at altitudes above 6,000 m while heat lost to evaporation and respiration increased by about 60 W⋅m^–2^ from 80 to 140 W⋅m^–2^, the net heat gain was about 40 W⋅m^–2^. Climbers need to understand the relationship between physical activity and heat balance. Situations that force a climber to stop or bivouac—such as fatigue, weather deterioration, sunset, or trauma—provoke a large decrease in metabolic heat production that is difficult to counteract in severe high-altitude conditions. Ainslie and Reilly (2003) suggested that the risk of hypothermia rises to critical levels when a fatigued mountaineer stops during descent or is forced to bivouac. The lower exertion that a climber requires on descent also reduces heat production, which falls precipitously if the climber stops (Ainslie and Reilly, 2003). Hypothermia also increases fatigue by reducing muscle strength and increasing oxygen consumption for the same intensity of exercise (Hinde et al., 2017). The message for a climber is: “If you stop, the hypothermia starts.”

### Respiration Heat Loss

The increase in respiration heat loss with altitude that we observed was less significant than the heat lost to convection or long-wave radiation. Respiration heat loss depends on the temperature of inhaled air, so any way of raising the temperature of inhaled atmospheric air or its mixture with oxygen from a container would decrease heat loss. Nevertheless, less advantage is gained from ameliorating respiration heat loss than from decreasing convective heat loss with proper clothing. Determining the ventilation level at any altitude is difficult, especially above 8,000 m where respiratory acidosis due to extreme hyperventilation determines the arterial partial pressure of oxygen necessary for survival (West et al., 2007a). Respiration level depends not only on activity level but also acclimatization, supplemental oxygen use and individual reaction to hypoxia. MENEX-2005 calculates respiration heat loss mainly on physical activity, so our results might underestimate the level of respiration heat loss, especially at extreme altitudes.

### Evaporative Heat Loss and Water Loss

The significant part of the total heat loss is due to evaporation, which increases with higher levels of physical activity. At an activity level of 5 MET with metabolic heat production of 290 W⋅m^–2^ evaporation was responsible for about 50% of the total heat lost at the altitudes of Phortse and Camp 2. By comparison, convection was responsible for less than 20% of the heat lost at those altitudes. Only at the altitude of South Col in winter in weak wind were evaporation and convective heat loss comparable, with each being responsible for 30–40% of total heat loss. Convective heat loss dominated at the altitude of South Col in windy winter conditions and was responsible for 50% of the total heat loss; evaporative heat loss accounted for 25%. Evaporation from the human body is generally influenced by metabolic heat production, air and skin temperature (which activate sweat glands), wind speed (which accelerates evaporation of sweat), and the clothing barrier (Błażejczyk and Kunert, 2011). Our research confirmed that high metabolic values dominated evaporation.

The amount of water a climber loses mostly depends on evaporation, which is mainly influenced by level of physical activity. Our calculations showed that alpinists lost about 2 liters of water to evaporation in 8 h of climbing at altitudes above 6,000 m. Substantial water was lost when considering the additional water lost through increased respiration. Climbers should drink 4–5 liters of fluids daily at altitude to maintain water equilibrium (Pollard and Murdoch, 1997).

### Solar Radiation Heat Gain

Despite the significant increase of global solar radiation and absorbed solar radiation with altitude, we found they played a negligible role in heat balance. The insulation of clothing used at high altitude decreased the role of absorbed solar radiation. The intense solar radiation we observed at high altitude warrants further research on developing clothing materials that would be able to store solar energy and return it as heat when needed.

### Limitations and Strengths

The MENEX-2005 model calculates respiration heat loss without considering the increase of ventilation due to hypoxia, which might therefore underestimate respiration heat loss, especially at extreme altitudes. We calculated metabolic heat production and levels of physical activity at different altitudes based on the daily energy expenditure of climbers observed by other authors. We understand that the values we assumed might be too general and over-simplified. The level of a climber’s physical activity depends on many variables, including the difficulty of the route, whether the climber is belaying or climbing, and the number of climbers on the route especially now that queues form on Everest. Yet the most important factor limiting climbers remains the level of oxygen, especially at extreme altitudes. Climbing speed and metabolic heat production change considerably while climbing. Our calculations did not range above 8,000 m, which is below the highest altitudes that climbers experience in the Himalayas.

This study provides the first complete assessment of human heat balance at altitudes between 4,000 and 8,000 m in active climbing. We based our calculations on *in situ* measurements rather than reanalysis, which provides only approximate values of weather factors. Our results should provide important practical benefits for climbers by helping them to better interpret weather forecasts and correctly choose the insulation properties of their clothing and emergency equipment.

Human heat balance at altitude needs further research. We concentrated on the active phase of climbing, but the cessation of movement puts climber at great risk of hypothermia. Further research is needed on the precise assessment of heat balance in climbers at rest during the static phase of climbing. The future task is also better representation of respiratory heat loss due to forced ventilation and reduced oxygen volume in the air.

## Conclusion

1. Alpinists face little risk of overheating or overcooling while actively climbing Mt. Everest, despite the potential risk of overcooling at high altitude on Mt. Everest in winter.
2. Convection and evaporation are responsible for most of the heat lost at altitude.
3. Levels of physical activity and clothing insulation play the greatest role in counteracting heat loss at high altitude.
4. Air pressure greatly influences convective heat loss, reducing its effect at higher altitudes.
5. The significant increase in solar radiation with altitude has little effect on the heat balance of climbers.
6. Respiration heat loss and heat balance in the static phase of climbing needs further research.

## Data Availability Statement

The original contributions presented in the study are included in the article/supplementary material, further inquiries can be directed to the corresponding author/s.

## Author Contributions

RS and KB contributed to conceptualization, methodology, validation, data curation, formal analysis, investigation, supervision, writing—original draft preparation, resources, visualization, writing—review, and editing. Both authors contributed to the article and approved the submitted version.

## Conflict of Interest

The authors declare that the research was conducted in the absence of any commercial or financial relationships that could be construed as a potential conflict of interest.

## Publisher’s Note

All claims expressed in this article are solely those of the authors and do not necessarily represent those of their affiliated organizations, or those of the publisher, the editors and the reviewers. Any product that may be evaluated in this article, or claim that may be made by its manufacturer, is not guaranteed or endorsed by the publisher.

## Annexure

### Radiation balance (Q)

Radiation balance (Q, W⋅m^–2^) is the sum of absorbed solar radiation (R) and net long-wave radiation (L):

(1)
Q=R+L

The SolGlob model developed by Błażejczyk (Błażejczyk, 1994, 2005a,b; Błażejczyk et al., 2012) based on empirical research was used to calculate the absorbed solar radiation. Given the information on the intensity of total solar radiation (Kglob), the formulas for calculating the value of R have various forms, depending on the height of the Sun (h) and the theoretically possible value of total radiation in a cloudless sky (Kt), which is calculated as follows:

(2)
$$\mathrm{Kt}=-0.0015\cdot h^{3}+0.1796\cdot h^{2}+9.6375\cdot h-11.9$$

The formulas for calculating the absorbed solar radiation have the following form:

- for h ≤ 12°.

(3)
$$R=\left(0.0014\cdot {\mathrm{Kglob}}^{2}+0.476\cdot \mathrm{Kglob}-3.8\right)\cdot \left(1-0.01\cdot \mathrm{ac}\right)\cdot \mathrm{Irc}$$

- for h > 12° and Kglob/Kt ≤ 0.8

(4)
$$R=0.247\cdot {\mathrm{Kglob}}^{0.9763}\cdot \left(1-0.01\cdot \mathrm{ac}\right)\cdot \mathrm{Irc}$$

- for h > 12° and Kglob/Kt from 0.81 to 1.05

(5)
$$R=3.692\cdot {\mathrm{Kglob}}^{0.5842}\cdot \left(1-0.01\cdot \mathrm{ac}\right)\cdot \mathrm{Irc}$$

- for h > 12° and Kglob/Kt from 1.06 to 1.2

(6)
$$R=43.426\cdot {\mathrm{Kglob}}^{0.2326}\cdot \left(1-0.01\cdot \mathrm{ac}\right)\cdot \mathrm{Irc}$$

- for h > 12° and Kglob/Kt > 1.2

(7)
$$R=8.928\cdot {\mathrm{Kglob}}^{0.4861}\cdot \left(1-0.01\cdot \mathrm{ac}\right)\cdot \mathrm{Irc}.$$

In the equations above, Irc is the dimensionless coefficient of the attenuation of heat flow through the clothing (for absorbed solar radiation, long-wave radiation and convection), which depends on coefficient of heat transfer by convection (hc) and coefficient of heat transfer by conduction within clothing (hc’). Irc is calculated as follows:

(8)
$$\mathrm{Irc}={\mathrm{hc}}^{\prime }/\left[{\mathrm{hc}}^{\prime }+\mathrm{hc}+21.55\cdot 10^{-8}\cdot {\left(\mathrm{Ta}+273\right)}^{3}\right]$$

(9)
$$\mathrm{hc}=\left(0.013\cdot \mathrm{ap}-0.04\cdot \mathrm{Ta}-0.503\right)\cdot {\left(v+v^{\prime }\right)}^{0.4}$$

(10)
$${\mathrm{hc}}^{\prime }=\left(0.013\cdot \mathrm{ap}-0.04\cdot \mathrm{Ta}-0.503\right)\cdot 0.53/\left\{\mathrm{Icl}\cdot \left[1-0.27\cdot {\left(v+v^{\prime }\right)}^{0.4}\right]\right\}$$

The long-wave (L) radiation balance consists of radiation emitted by the surface of the body/clothing (Ls) and thermal radiation emitted by the surface (Lg) and the reverse radiation of the atmosphere (La):

(11)
L=(0.5⋅Lg+0.5⋅La-Ls)⋅Irc

(12)
$$\mathrm{where}:\mathrm{Ls}=5.38\cdot 10^{-8}\cdot {\left(273+\mathrm{Tsk}\right)}^{4}.$$

The skin temperature (Tsk) is calculated from the empirical formula below:

(13)
Tsk=(26.4+0.0214⋅Mrt+0.2095⋅Ta-0.018⋅RH-0.01⋅v)+0.6⋅(Icl-1)+0.00128⋅M

where: mean radiant temperature (Mrt) is calculated as follows:

(14)
$$\mathrm{Mrt}={\left[\left(R/\mathrm{Irc}+\mathrm{Lg}+\mathrm{La}\right)/\left(5.38\cdot 10^{-8}\right)\right]}^{0.25}-273$$

### Evaporative Heat Loss (E)

Evaporative heat loss (E, W⋅m^–2^) depends on the difference in water vapor pressure on the surface of the skin (vps) and in the ambient air (vp) and coefficient of heat transfer by evaporation (he). The degree of skin moisture (w), metabolic heat production (M) and dimensionless coefficient of the attenuation of heat flow through the clothing by evaporation (Ie) are also taken into account:

(15)
E=he⋅(vp-vps)⋅w⋅Ie-[0.42⋅(M-58)-5.04]

(16)
$$\mathrm{where}:\mathrm{vps}=e^{\left(0.058\cdot \mathrm{Tsk}+2.003\right)}$$

(17)
w=1.031/(37.5-Tsk)-0.065

(18)
$$\mathrm{he}=\left[\mathrm{Ta}\cdot \left(0.00006\cdot \mathrm{Ta}-0.00002\cdot \mathrm{ap}+0.011\right)+0.02\cdot \mathrm{ap}-0.773\right)]\cdot 0.53/\{\mathrm{Icl}\cdot \left[1-0.27\cdot {\left(v+v^{\prime }\right)}^{0.4}\right]\}$$

(19)
$$\mathrm{Ie}={\mathrm{hc}}^{\prime }/\left({\mathrm{hc}}^{\prime }+\mathrm{hc}\right)$$

The information on evaporative heat loss is supplemented by the determination of water loss due to sweating (SW, in g⋅h^–1^). Its calculation is based on the potential values of evaporative heat loss (Epot). Epot is derived from the MENEX_2005 model taking into account 5% level of relative humidity of air (RH):

(20)
SW=-2.6⋅Epot

where Epot is calculated the same way as E (see equation 15) but with vp replaced by vp’. vp’ is vapor pressure equal to 5% RH as follows:

(21)
$${\mathrm{vp}}^{\prime }=6.112\cdot 10^{\left[7.5\cdot \mathrm{Ta}/\left(237.7+\mathrm{Ta}\right)\right]}\cdot 0.05$$

### Convective Heat Exchange (C)

Convective heat exchange (C, W⋅m^–2^) depends on the difference between the average skin temperature (Tsk) and the air temperature (Ta), on the speed of air movement, and on its density and heat capacity (these variables are included in the coefficient hc):

(22)
C=hc⋅(Ta-Tsk)⋅Irc

### Respiration Heat Loss (Res)

Respiration heat loss (Res, W⋅m^–2^) depends on the difference between the air temperature (Ta) and the exhaled air temperature (it is assumed to be 35.0°C) and on the difference between the water vapor pressure in the atmosphere (vp) and the water vapor pressure in the exhaled air (equal to 56.24 hPa):

(23)
Res=0.0014⋅M⋅(Ta-35)+0.0173⋅M⋅[0.1⋅(vp-56.24)].
